# Genome-wide association study of reproductive traits in Nellore heifers using Bayesian inference

**DOI:** 10.1186/s12711-015-0146-0

**Published:** 2015-08-19

**Authors:** Raphael B. Costa, Gregório MF Camargo, Iara DPS Diaz, Natalia Irano, Marina M. Dias, Roberto Carvalheiro, Arione A. Boligon, Fernando Baldi, Henrique N. Oliveira, Humberto Tonhati, Lucia G. Albuquerque

**Affiliations:** UNESP, Universidade Estadual Paulista, Faculdade de Ciências Agrárias e Veterinárias, Jaboticabal, 14884-900 São Paulo Brazil

## Abstract

**Background:**

An important goal of Zebu breeding programs is to improve reproductive performance. A major problem faced with the genetic improvement of reproductive traits is that recording the time for an animal to reach sexual maturity is costly. Another issue is that accurate estimates of breeding values are obtained only a long time after the young bulls have gone through selection. An alternative to overcome these problems is to use traits that are indicators of the reproductive efficiency of the herd and are easier to measure, such as age at first calving. Another problem is that heifers that have conceived once may fail to conceive in the next breeding season, which increases production costs. Thus, increasing heifer’s rebreeding rates should improve the economic efficiency of the herd. Response to selection for these traits tends to be slow, since they have a low heritability and phenotypic information is provided only later in the life of the animal. Genome-wide association studies (GWAS) are useful to investigate the genetic mechanisms that underlie these traits by identifying the genes and metabolic pathways involved.

**Results:**

Data from 1853 females belonging to the Agricultural Jacarezinho LTDA were used. Genotyping was performed using the BovineHD BeadChip (777 962 single nucleotide polymorphisms (SNPs)) according to the protocol of Illumina - Infinium Assay II ® Multi-Sample HiScan with the unit SQ ™ System. After quality control, 305 348 SNPs were used for GWAS. Forty-two and 19 SNPs had a Bayes factor greater than 150 for heifer rebreeding and age at first calving, respectively. All significant SNPs for age at first calving were significant for heifer rebreeding. These 42 SNPs were next or within 35 genes that were distributed over 18 chromosomes and comprised 27 protein-encoding genes, six pseudogenes and two miscellaneous noncoding RNAs.

**Conclusions:**

The use of Bayes factor to determine the significance of SNPs allowed us to identify two sets of 42 and 19 significant SNPs for heifer rebreeding and age at first calving, respectively, which explain 11.35 % and 6.42 % of their phenotypic variance, respectively. These SNPs provide relevant information to help elucidate which genes affect these traits.

## Background

In tropical regions, Zebu cattle are the main breed used for beef production since they are better adapted to the climate and more resistant to parasites than taurine breeds. One factor that increases production costs is the late onset of reproduction in Zebu females. According to Brummati et al. [1], for beef cattle that are raised in tropical production systems, reproductive traits possess a high economic value and are up to 13 times economically more important than growth and carcass traits. Advancing the start of the reproductive cycle brings economic benefits to the producer and increases profitability.

In Brazil, beef cattle breeding programs have put emphasis mainly on traits such as growth and weight gain and little attention has been paid to reproductive traits, in spite of the considerable influence that these traits have on the productivity and reproductive efficiency of herds [2–4]. According to Evans [5], females of breeding age account for about 70 % of the costs of the beef cattle production system.

Heifer rebreeding (HR) refers to the calving success of cows that have already calved once. The success rate of rebreeding after first calving is a major issue in beef cattle farming. Several studies have reported significant reductions in calving rates between the first and second services [6–8]. Mercadante et al. [9] observed reductions of up to 20 % in calving rates from the first to the second conception of Nellore females. Therefore, improving the rebreeding rate of heifers should increase the economic efficiency of beef cattle production.

Since age at first calving (AFC) is easily measured and is a good indicator of female sexual precocity, this trait has been included as a selection objective in breeding programs [10]. However, direct selection for lower AFC is not simple since some producers delay the entry of females into breeding programs, which determines the age or weight at which reproductive activity begins and impairs the identification of sexually precocious females. Furthermore, the heritability estimates of this trait are generally low to moderate (0.09 to 0.28) [2, 10–12], which suggests that response to selection for AFC is a slow process.

Female reproductive traits are measured only on females and late in life, and generally have low heritability. As a consequence, a large number of individuals need to be analyzed for sufficiently accurate genetic evaluations. The advantage of genomic selection to estimate such reproductive traits is that, unlike traditional selection based on pedigree information and phenotypes only, it allows to select animals accurately without their own phenotypic measurements or those of their relatives and thus, genetic gains are increased compared to that obtained with traditional evaluation methods [13].

Usually, genome-wide association studies (GWAS) of traits of economic interest using high-density single nucleotide polymorphism (SNP) panels aim at identifying regions that can explain the inheritance of the traits [14]. GWAS results allow the identification of candidate regions that can then be used for genomic selection.

GWAS were first performed using the least square method, by applying Bonferroni correction to make inferences about the significance of individual SNPs. According to Peters [15], the main problems that are encountered with this method are high rates of false positives and overestimation of the effects of quantitative trait loci (QTL) and SNPs. One alternative to overcome these problems is to estimate SNP effects by Bayesian inference for which all SNPs are considered simultaneously.

The objective of this study was to perform a GWAS using a Bayesian approach to identify candidate genes that influence reproductive traits. Candidate genes are the target for future fine-mapping studies to search for causal mutations. Causal mutations that explain more than 1 % of the phenotypic variance are useful for inclusion in commercial low-density SNP arrays because since they are causal and are not in linkage disequilibrium (LD) with nearby SNPs, they are more cost-effective and informative, contribute information for the estimation of breeding values and it is not necessary to revalidate their SNP effects at each generation.

## Methods

### Phenotypic data

We used data from Nellore females that belonged to the company Agropecuária Jacarezinho Ltda. Heifers were born either in Valparaiso-SP or in Cotegipe-BA, Brazil. *Bos indicus* reaches puberty later in life than *Bos taurus*. In order to detect sexually precocious heifers, Agropecuária Jacarezinho Ltda. performs two breeding seasons. During the early breeding season, which occurs between February and April and lasts approximately 60 days, heifers are exposed to bulls at an early age (14 to 16 months). All heifers are exposed to bulls, irrespective of weight and body condition. Artificial insemination, controlled breeding or multiple-sire breeding, with a bull:cow ratio of 1:30, is used. Pregnancy is confirmed about 60 days after the end of the anticipated breeding season. Heifers that did not conceive during their first breeding season are exposed again to bulls at 2 years of age. The criteria for culling of females are: failure to conceive before 2 years of age, inability to conceive in any subsequent year, and low progeny performance. A small percentage of females are culled due to health problems. Close to the expected calving date, females are moved to calving paddocks. After calving, calves are divided according to sex and age and are transferred to another pasture together with their mothers where they remain until weaning. The calves are weaned at 7 months and grouped together until yearling age (18 months).

Heifer rebreeding (HR) is a binary trait and was defined by attributing a value of 2 (success) or 1 (failure) to females that calved or not, respectively, given that they had calved once. Age at first calving, measured in days, was obtained as the difference between the date of first calving and the date of birth of the heifer.

The contemporary group (CG) for HR was defined by farm, year and season of birth of the cow, and calf sex. Contemporary groups without variability in HR, i.e., groups in which all animals showed the same response category (1 or 2) were eliminated. For AFC, the CG was formed by farm, year and season of birth, and management group at birth, weaning and yearling. Phenotypic data outside the intervals given by the mean of the CG ± 3 standard deviations were also excluded. Fifteen CG were formed for HR with an average of 92 animals in each group, and 17 CG were formed for AFC with an average of 89 animals in each group. Age at first calving varied between 748 and 1253 days, with a mean of 1049 ± 141.3 days. The percentage of success in HR was equal to 72,42 % ± 19,21.

### Genotypic data

Data from 2056 females born between 2007 and 2009, which were genotyped with the Illumina Bovine HD assay (Illumina, San Diego, CA, USA), were used. The quality control (QC) of genotypes was performed iteratively according to the following criteria: we excluded 22 851 SNPs in non-autosomal regions, 32 856 SNPs with a gene call score less than 0.70, 18 982 SNPs with a call rate less than 0.98, 362 148 SNPs with a minor allele frequency (MAF) less than 0.02, 9466 SNPs with a p-value for the Hardy-Weinberg equilibrium test less than 10^−5^ and 26 341 SNPs that were highly correlated (r^2^ > 0.995) with other SNPs from the same window that contained 100 consecutive SNPs. Samples with a call rate less than 0.90 were also excluded from the analysis. The QC process was repeated until no further SNP or sample was excluded which resulted in a final dataset of 1853 heifer and 305 348 SNPs.

### Data analysis

SNP effects were estimated using the BAYESCπ method [16] in which they have a common variance, follow a scaled inverted chi-square distribution *a priori*, with ν_g_ degrees of freedom and a scale parameter *S*_g_^2^. Thus, the effect of an SNP with probability (1-π) is a univariate Student’s *t* (0, ν_g,_*S*_g_^2^) distribution. In this study, ν_g_ was set to 4.2 and *S*_g_^2^ was calculated based on additive genetic variance according to Habier et al. [17].

The analyses were performed using the GS3 software developed by Legarra et al. (2011; http://snp.tolouse.inra.fr/~alegarra). A total of 300 000 MCMC iterations were used, with a burn-in period of 30 000 cycles and the results were saved every 30 cycles. Convergence was assessed by visual inspection of trace plots of the posterior density of genetic and residual variances.

The dependent variables used in the analysis were the phenotypes observed for HR and AFC. All females with available genotypes and phenotypes were used. The following model was applied:

$$ \mathbf{y}\kern0.5em =\kern0.5em \mathbf{X}\mathbf{b}\kern0.5em +\kern0.5em \mathbf{Z}\mathbf{u}\kern0.5em +\kern0.5em \mathbf{W}\mathbf{g}\kern0.5em +\kern0.5em \mathbf{e}, $$

where **y** is a vector of phenotypes; **X** is an incidence matrix of systematic effects; **b** is a vector of systematic effects; **Z** is an incidence matrix of polygenic effects; **u** is a random vector of polygenic effects of all individuals in the pedigree; **W** is a matrix (n x s) consisting of the genotypes of s SNPs for each animal *n*; **g** is a random vector of SNP effects; and **e** is a vector of residual effects. A systematic effect of CG was assumed for each trait. For HR, the linear effect of the rest period (number of postpartum days until the beginning of the second breeding season) was included as a covariate. An inverted chi-square distribution with 4.2° of freedom was assumed for the *a priori* distribution of residual variance.

It is not possible to obtain p-values for the SNP effects using Bayesian approaches. An alternative to p-values is the Bayes factor [16], which was calculated to evaluate the significance of the SNPs on the traits as follows:

$$ BF\kern0.5em =\frac{\left({\scriptscriptstyle \frac{p}{1\kern0.5em -\kern0.5em p}}\right)}{\left({\scriptscriptstyle \frac{\pi }{1\kern0.5em -\kern0.5em \pi }}\right)}, $$

where *p* is the posterior probability of an SNP to be assigned a non-zero effect and π is the *a priori* probability of an SNP to be included in the analysis. The following scale adapted by Kass and Raftery [18] and applied in QTL detection by Varona et al. [16] and Vidal et al. [19] was used:

- if BF are between 3 and 20, they provide suggestive evidence;
- if BF are between 20 and 150, they provide strong evidence;
- and if BF are greater than 150, they provide very strong evidence.

When BF are used, there is no need for Bonferroni correction because all the SNPs are introduced simultaneously in the analysis, and their estimates are already penalized by their prior information. In this study, SNPs with a BF greater than 150 were considered significant.

### Identification of genes

The Map Viewer tool of the bovine genome (http://www.ncbi.nlm.nih.gov/projects/mapview/map_search.cgi?taxid=9913&build=6) was used to determine the location of the significant SNPs on the genome. Genes that contained significant SNPs were listed. For SNPs that were not located within genes, the closest gene (either on the 5’ or 3’ end) was recorded with the distance between gene and SNP.

## Results and discussion

**Fig. 1:**
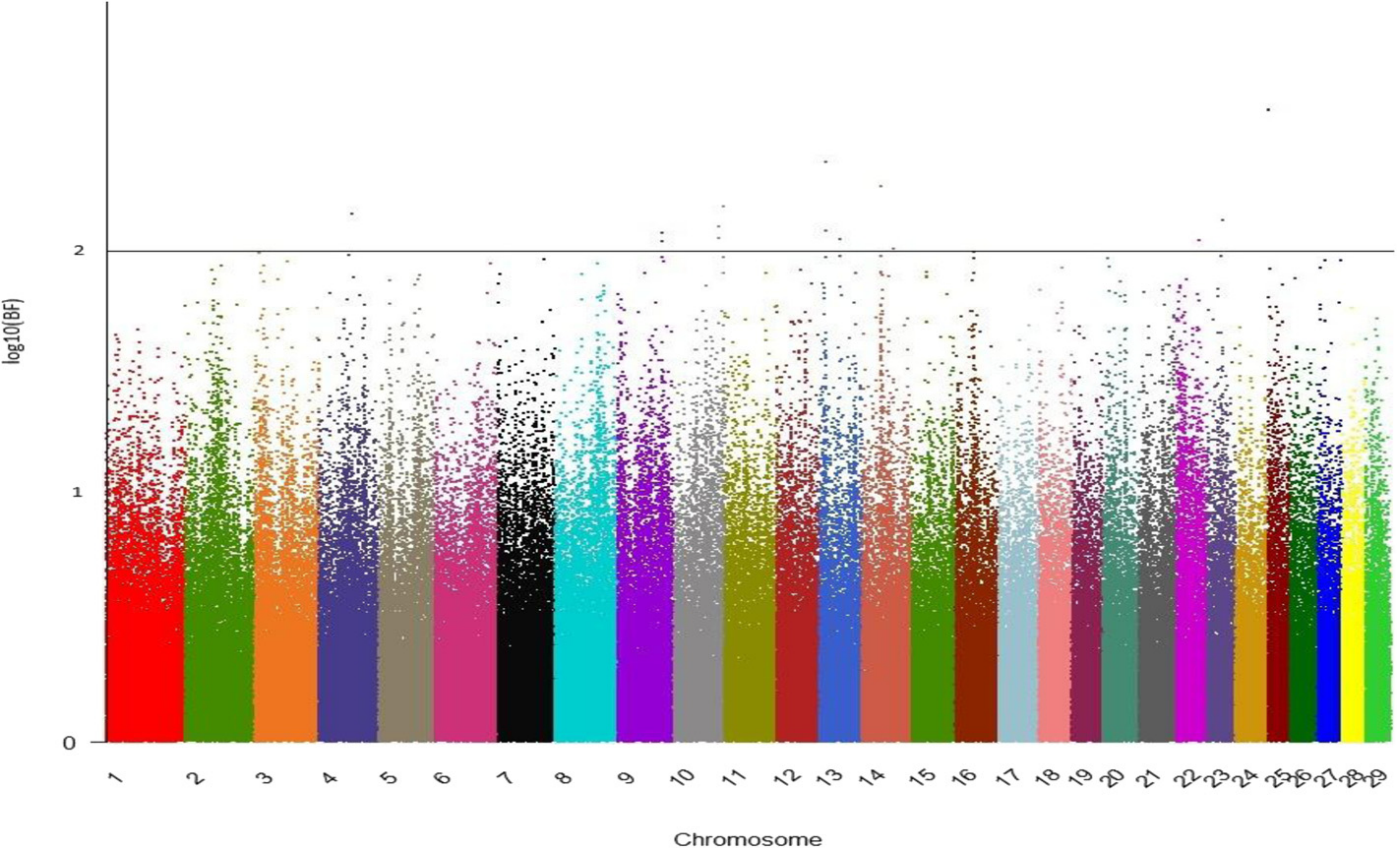
Manhattan plot for age at first calving. The y- and x-axes indicate the logarithm (base 10) of Bayes factor and chromosome number, respectively

**Fig. 2:**
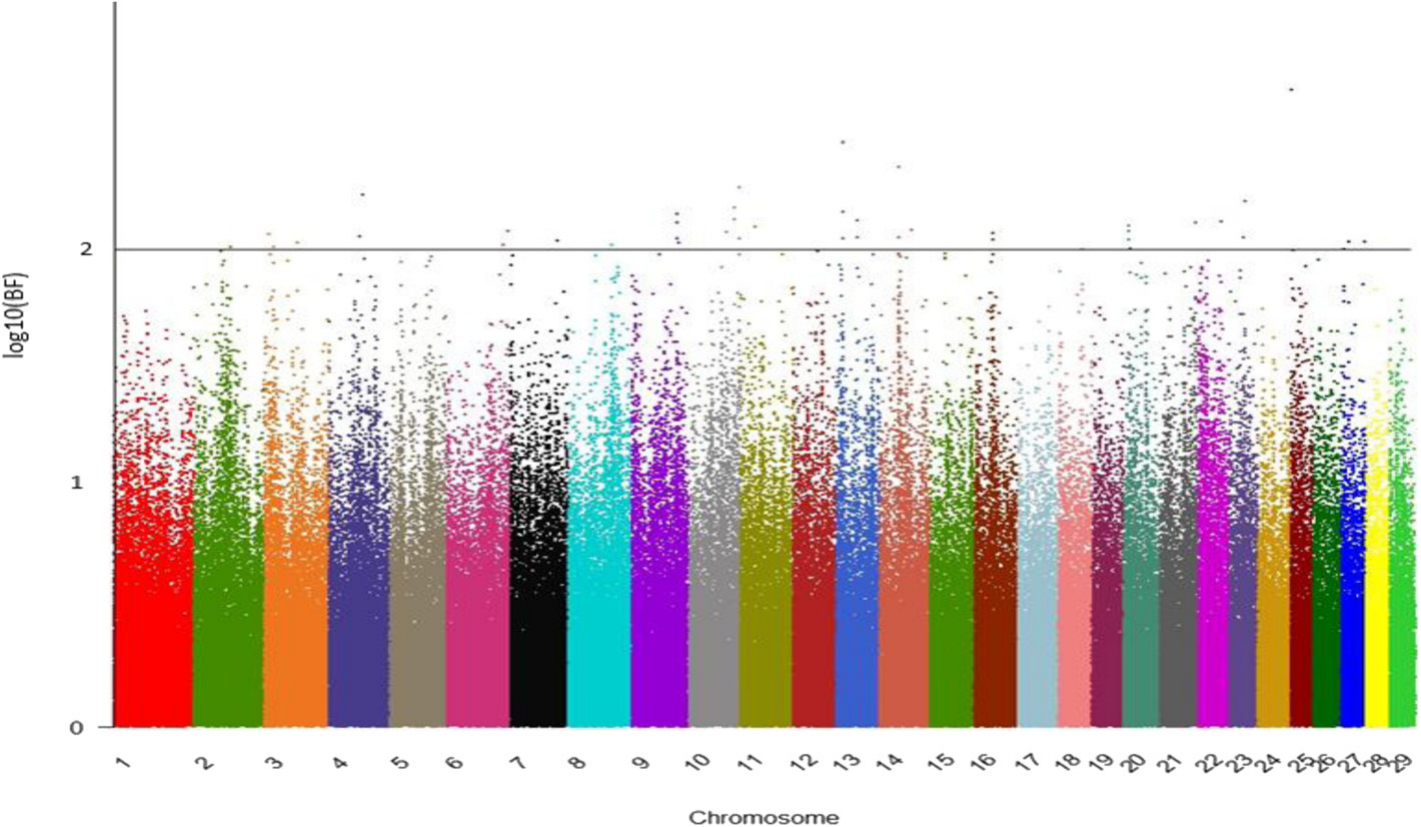
Manhattan plot for heifer rebreeding. The y- and x-axes indicate the logarithm (base 10) of Bayes factor and chromosome number, respectively

Forty-two and 19 SNPs with a Bayes factor greater than 150 were detected for HR and AFC, respectively (Figs. 1 and 2). As expected, all SNPs that were significant for AFC were also significant for HR (Table 1), since both traits are indicators of reproductive efficiency in beef cattle and should be, at least in part, under the control of the same groups of genes. Gene symbols and their respective names are in Table 2.

**Table 1:** Significant SNPs for heifer rebreeding and age at first calving

SNP	BTA	Position on the chromosome	Gene	Distance
BovineHD0200019275	2	66 618 544	*LOC781274*	32 131
BovineHD0300002153	3	6 820 16	*DDR2*	0
BovineHD0300004387	3	13 529 885	*LOC787257*	97 632
BovineHD0300017549	3	58 324 511	*COL24A1*	0
BovineHD0400035711	4	65 529 242	*NEUROD6*	36 652
BovineHD0400020154^a^	4	72 761 718	*ADAM22*	0
BovineHD0700027122	7	92 796 302	*GPR98*	0
BovineHD0800023325	8	77 860 555	*FRMD3*	32 334
BovineHD0900024229^a^	9	86 427 969	*LOC784274*	58 945
BovineHD0900024233^a^	9	86 443 282	*LOC784274*	74 258
BovineHD0900024387^a^	9	86 9312 92	*SASH1*	0
BovineHD0900024630^a^	9	87 622 468	*LOC100847971*	132 138
BovineHD1000022473	10	78 547 946	*LOC785763*	83 898
BovineHD1000027195^a^	10	94 149 370	*SEL1L*	184 234
BovineHD1000027196^a^	10	94 150 213	*SEL1L*	185 077
BovineHD1000029149^a^	10	100 682 598	*GALC*	50 483
BovineHD1000029156	10	100 692 866	*GALC*	40 215
BovineHD1100007588	11	25 331 444	*HAAO*	129 881
BovineHD1300002848^a^	13	10 469 479	*KIF16B*	230 839
BovineHD1300003397^a^	13	12 043 707	*CAMK1D*	83 121
BovineHD1300003401	13	12 047 548	*CAMK1D*	86 962
BovineHD1300010715^a^	13	37 037 785	*ARMC4*	0
BovineHD1300010716	13	37 041 136	*ARMC4*	0
BovineHD1400008910^a^	14	30 847 011	*MIR124A*-*2*	31 490
BovineHD1400008946^a^	14	30 992 834	*CYP7B1*	0
BovineHD1400015365^a^	14	54 735 529	*LOC782102*	1 572 261
BovineHD1600009851	16	34 426 540	*SDCCAG8*	0
BovineHD1600009861	16	34 439 744	*SDCCAG8*	0
BovineHD1800012552	18	42 481 407	*LOC783434*	109 417
BovineHD2000003208^a^	20	10 148 419	*GTF2H2*	0
BovineHD2000003210	20	10 153 352	*OCLN*	1 557
BovineHD2000003214	20	10 160 002	*OCLN*	0
BovineHD2000003968	20	12 416 566	*LOC529061*	0
BovineHD2100018913	21	64 294 259	*LOC100847341*	592 456
BovineHD2200012027^a^	22	41 764 132	*FHIT*	0
BovineHD2300006979	23	25 155 638	*ELOVL5*	104
BovineHD2300008276^a^	23	29 144 150	*LOC514434*	0
BovineHD2500000286^a^	25	1 323 539	*MAPK8IP3*	0
BovineHD2500001186^a^	25	5 120 120	*RBFOX1*	297 144
BovineHD2700001047	27	3 081 686	*LOC782601*	69 309
BovineHD2700003551	27	12 300 159	*ODZ3*	56 728
BovineHD2700012809	27	44 150 097	*ZNF385D*	0

^a^indicates SNPs that are shared by both traits

**Table 2:** Gene symbols and full names

Gene symbol	Gene name
*LOC781274*	*protein O*-*mannose kinase*-*like*
*DDR2*	*discoidin domain receptor tyrosine kinase 2*
*LOC787257*	*rab GDP dissociation inhibitor beta*-*like*
*COL24A1*	*collagen*, *type XXIV*, *alpha 1*
*NEUROD6*	*neuronal differentiation 6*
*ADAM22*	*ADAM metallopeptidase domain 22*
*GPR98*	*G protein*-*coupled receptor 98*
*FRMD3*	*FERM domain containing 3*
*LOC784274*	*kidney mitochondrial carrier protein 1*
*SASH1*	*SAM and SH3 domain containing 1*
*LOC100847971*	*60S ribosomal protein L30*-*like*
*LOC785763*	*2*,*3*-*bisphosphoglycerate mutase pseudogene*
*SEL1L*	*sel*-*1 suppressor of lin*-*12*-*like*
*GALC*	*galactosylceramidase*
*HAAO*	*3*-*hydroxyanthranilate 3*,*4*-*dioxygenase*
*KIF16B*	*kinesin family member 16B*
*CAMK1D*	*calcium*/*calmodulin*-*dependent protein kinase ID*
*ARMC4*	*armadillo repeat containing 4*
*MIR124A*-*2*	*microRNA mir*-*124a*-*2*
*CYP7B1*	*cytochrome P450*, *family 7*, *subfamily B*, *polypeptide 1*
*LOC782102*	*UDP*-*GlcNAc*:*betaGal beta*-*1*,*3*-*N*-*acetylglucosaminyltransferase 2*
*SDCCAG8*	*serologically defined colon cancer antigen 8*
*LOC783434*	*serine*/*threonine*-*protein kinase pim*-*1 pseudogene*
*GTF2H2*	*general transcription factor IIH*, *polypeptide 2*
*OCLN*	*occludin*
*LOC529061*	*microtubule associated serine*/*threonine kinase family member 4*
*LOC100847341*	*uncharacterized LOC100847341*
*FHIT*	*fragile histidine triad*
*ELOVL5*	*ELOVL fatty acid elongase 5*
*LOC514434*	*olfactory receptor 5 V1*
*MAPK8IP3*	*mitogen*-*activated protein kinase 8 interacting protein 3*
*RBFOX1*	*RNA binding protein*, *fox*-*1 homolog*
*LOC782601*	*histone H3-like centromeric protein A*
*ODZ3*	*teneurin transmembrane protein 3*
*ZNF385D*	*zinc finger protein 385D*

The 42 SNPs that were significant for HR are located within or next to 35 genes that are distributed over 18 chromosomes; of these, 27 are protein-encoding genes, six are pseudogenes, and two are miscellaneous noncoding (nc) RNAs. A pseudogene is a nucleotide sequence that is similar to a normal gene, but is not expressed. Miscellaneous nc RNAs are small noncoding RNAs sequences that do not carry information for producing proteins but can have various important functions in the cell.

Genes that contained significant SNPs were listed (distance = 0); if the SNPs were not located within genes, the closest gene (either on the 5’ or 3’ end) was identified with the distance between SNP and gene. Espigolan et al. [20] reported that since LD in Nellore cattle was lower than in *Bos taurus* breeds, a distance of less than 30 kb was required for genomic prediction/association. However, in our study, although some genes were more than 30 kb away from a significant SNP, they were retained because they were the closest annotated genes.

The identified genes can be grouped according to metabolic function, including groups of genes that are involved in the formation and physiology of the central nervous system (*LOC781274*, *NEUROD6*, *GPR98*, *GALC*, *MIR124A*-*2*, *MAPK8IP3*, *RBFOX1*, and *ODZ3*); in the formation and physiology of the female reproductive system (*FMRD3*, *CYP7B1*, and *LOC782102*); in lipid metabolism (*ELOVOL5*) and in bone growth (*DDR2* and *COL24A1*); genes that act as olfactory receptors (*LOC514434*) and genes involved in basal metabolism (*LOC787257*, *ADAM22*, *LOC100295124*, *LOC100847971*, *LOC785763*, *SEL1L*, *HAAO*, *KIF16B*, *CAMK1D*, *ARMC4*, *SDCCAG8*, *LOC783434*, *GTF2H2*, *OCLN*, *LOC529061*, *LOC529061*, *FHIT*, and *LOC782601*). The last group of genes plays a role in different metabolic pathways, including cell-cell signaling, protein synthesis and transport, oxygen transport, cell proliferation and survival, transcription and metabolism of nucleotides and histones, membrane transport, and the formation of the cell membrane and its receptors, among others.

Genes that are involved in the formation and physiology of the central nervous system play a role in reproduction, since they influence neuronal formation, differentiation and communication and the synthesis of reproductive hormones of the hypothalamus-pituitary axis by acting on the hormone cascade that coordinates the estrous cycle in females. Thus, because the genomic regions that are described here are biologically relevant to animal physiology, they are good candidates for marker-assisted selection. Fortes et al. [21–23] and Hawken et al. [24] identified other genes that belong to the same group, i.e., genes acting on the central nervous system, associated with puberty and fertility traits in Brahman cattle and tropical composite breeds.

Similarly, genes that are involved in the formation and physiology of the female reproductive system should affect the onset of the estrous cycle, conception, pregnancy establishment and maintenance, and calving. In general, the genes of this group that were detected here act on the formation of specific tissues and hormone synthesis. One example is the *LOC782102* gene which encodes a component of the egg membrane that is responsible for sperm attraction. Polymorphisms in *LOC782102* may result in a protein that is more or less functional for spermatozoid recognition and thus pregnancy will be either facilitated or impaired.

Lipid metabolism is intimately related to reproduction and according to [25], in dairy cattle, the success of postpartum rebreeding depends on the accumulation of fat reserves in the animal, which permits the cow to start cycling again. During the postpartum period, dairy cows enter a state of negative energy balance and mobilize body fat for milk production since they are unable to meet their energy requirement solely through feeding. As a consequence, the presence of favorable alleles of genes related to energy metabolism may be associated with better reproductive performance. Many genes related to fat metabolism and, consequently, to reproduction have been described in dairy cattle [26, 27]. This mechanism has already been demonstrated in beef cattle and genes that are part of this metabolic pathway have been reported [21].

It is known that growth influences reproduction as demonstrated by the observation that heavier animals reach sexual maturity later in life [28]. Growth-related genes have been associated with fertility and puberty in Zebu and tropical composite breeds [23, 24], in agreement with the results obtained here for genes related to bone growth.

Our results on the chromosome location of the identified SNPs are similar to those of previous investigations on the association between SNPs and reproductive traits. Sahana et al. [29] detected SNPs for fertility traits on most of the chromosomes in Danish and Swedish Holstein females, which were associated with pregnancy rate, interval from first to last insemination in cows, number of inseminations per conception in cows, and interval from calving to first insemination. The authors reported the presence of significant SNPs in a region between 28.5 and 68.06 Mb on BTA13 (BTA for *Bos taurus* chromosome), which is larger than the region that we identified here. However, the SNPs that we found significant for AFC and HR are within the region reported in [29].

Schulman et al. [30] identified significant SNPs on BTA27 between 6.08 and 21.65 Mb, which were associated with non-return rate for heifers in Finnish Ayrshire heifers. Here, we detected three significant SNPs on BTA27 in the region between 3.08 and 44.15 Mb, which, although much larger than that reported in [30], contains an SNP at 12.3 Mb very close to the SNP identified by [30], i.e., at 11.2 Mb. This result suggests the presence of a QTL on this chromosome.

Pausch et al. [31] described three significant SNPs for calving ease on BTA21 in Fleckvieh females at 2.15, 2.33, and 2.38 Mb. Although we also detected one SNP on BTA21, it is located at quite a large distance from those reported by Pausch et al. [31].

Hawken et al. [24] reported 66 significant SNPs (P < 0.001) for postpartum anestrous interval in Brahman cattle. Although their results showed that BTA3 and 14 contained the largest numbers of SNPs, the most significant SNP was on BTA6 at 118 Mb. In our case, we found no significant SNP on BTA6. The same authors also described 68 significant SNPs for first postpartum ovulation before weaning mainly on BTA3, 6, 14, 17 and 21 in Brahman cattle. The most significant SNP was at 112.3 Mb on BTA3. In our case, three significant SNPs were detected on BTA3, but in the region between 6.8 and 58.3 Mb.

Although several studies have reported significant SNPs associated with fertility traits in cattle on BTA14 [23, 24, 29–31], we did not detect any significant SNP associated with HR and AFC on this chromosome. This may be due to genetic differences between *Bos taurus* and *Bos indicus*, since *Bos indicus* females reach puberty later than *Bos taurus* females. Evidence from Hawken et al. [23], who reported that the number of significant SNPs on BTA14 associated with fertility traits was much smaller in Brahman cattle than that from other studies in *Bos taurus* [29–31], supports this hypothesis.

Taken together, the 42 SNPs significant for HR and the 19 SNPs significant for AFC explained 11.35 % (Table 3) and 6.42 % (Table 4) of the phenotypic variance of these traits, respectively. These SNPs will be useful to generate a specific panel for Nellore animals.

**Table 3:** Significant SNPs for heifer rebreeding with chromosome number, percentage of phenotypic variance explained by the SNP (%PV) and cumulative percentage (%CPV)

SNP	BTA	%PV	%CPV
BovineHD1000022473	10	1.45	1.45
BovineHD0900024233	9	1.25	2.70
BovineHD1400008910	14	1.08	3.78
BovineHD0300017549	3	1.02	4.80
BovineHD1600009861	16	0.69	5.49
BovineHD1000029156	10	0.55	6.04
BovineHD1300002848	13	0.37	6.41
BovineHD1300010716	13	0.37	6.78
BovineHD2500001186	25	0.35	7.13
BovineHD1400015365	14	0.34	7.47
BovineHD2700003551	27	0.31	7.78
BovineHD1600009851	16	0.3	8.08
BovineHD2000003210	20	0.27	8.35
BovineHD2200012027	22	0.26	8.61
BovineHD2300006979	23	0.25	8.86
BovineHD2000003214	20	0.24	9.10
BovineHD1300003397	13	0.22	9.32
BovineHD1300010715	13	0.22	9.54
BovineHD2700012809	27	0.21	9.75
BovineHD1400008946	14	0.19	9.94
BovineHD0900024387	9	0.17	10.11
BovineHD1300003401	13	0.16	10.27
BovineHD0900024630	9	0.15	10.42
BovineHD2000003208	20	0.15	10.57
BovineHD2700001047	27	0.09	10.66
BovineHD0800023325	8	0.08	10.74
BovineHD0900024229	9	0.08	10.82
BovineHD1000029149	10	0.08	10.9
BovineHD0700027122	7	0.07	10.97
BovineHD0300004387	3	0.05	11.02
BovineHD0400020154	4	0.05	11.07
BovineHD1000027195	10	0.05	11.12
BovineHD1800012552	18	0.05	11.17
BovineHD1000027196	10	0.04	11.21
BovineHD2500000286	25	0.04	11.25
BovineHD0200019275	2	0.03	11.28
BovineHD2000003968	20	0.03	11.31
BovineHD1100007588	11	0.02	11.33
BovineHD0300002153	3	0.01	11.34
BovineHD0400035711	4	0.01	11.35
BovineHD2100018913	21	0	11.35
BovineHD2300008276	23	0	11.35

**Table 4:** Significant SNPs for age at first calving with chromosome number, percentage of phenotypic variance explained by the SNP (%PV) and cumulative percentage (%CPV)

SNP	BTA	%PV	%CPV
BovineHD0900024233	9	1.02	1.02
BovineHD1400008910	14	0.82	1.84
BovineHD2500001186	25	0.82	2.66
BovineHD1000029149	10	0.61	3.27
BovineHD0900024630	9	0.52	3.79
BovineHD1300010715	13	0.51	4.30
BovineHD2500000286	25	0.37	4.67
BovineHD1300003397	13	0.25	4.92
BovineHD2000003208	20	0.22	5.14
BovineHD0400020154	4	0.21	5.35
BovineHD1000027195	10	0.21	5.56
BovineHD0900024229	9	0.15	5.71
BovineHD1300002848	13	0.14	5.85
BovineHD1400015365	14	0.14	5.99
BovineHD2200012027	22	0.12	6.11
BovineHD2300008276	23	0.09	6.20
BovineHD1400008946	14	0.08	6.28
BovineHD0900024387	9	0.07	6.35
BovineHD1000027196	10	0.07	6.42

## Conclusions

The use of Bayes factors to determine the significance of SNPs allowed us to identify two sets of significant SNPs, i.e., 42 for HR and 19 for AFC that explain 11.35 % and 6.42 % of their phenotypic variance, respectively. These SNPs provide relevant information about HR and AFC that will contribute to elucidate which genes affect these traits. Our results led us to suggest a list of candidate genes for reproductive traits in beef cattle.

## References

[1] Brumatti RC, Ferraz JBS, Eler JP, Formigonni IB (2011). Desenvolvimento de índices de seleção em gado de corte sob enfoque de um modelo bioeconômico. Arch Zootec..

[2] Boligon AA, Albuquerque LG (2011). Genetic parameters and relationships of heifer pregnancy and age at first calving with weight gain, yearling and mature weight in Nellore cattle. Livest Sci..

[3] Formigoni IB, Ferraz JBS, Silva JAIIV, Eler JP, Brumatti RC (2005). Valores econômicos para habilidade de permanência e probabilidade de prenhez aos 14 meses em bovinos de corte. Arq Bras Med Vet Zootec..

[4] Shiotsuki L, Silva JAV, Tonhati H, Albuquerque LG (2009). Genetic associations of sexual precocity with growth traits and visual scores of conformation, finishing, and muscling in Nellore cattle. J Anim Sci.

[5] Evans ACO (2003). Ovarian follicle growth and consequences for fertility in sheep. Anim Reprod Sci..

[6] Fahmy MH, Lalande G, Hidiroglou M (1971). Reproductive performance and growth of Shorthorn purebred and crossbred cows. Anim Prod..

[7] Rovira J (1974). Reproduccion y manejo de los rodeos de cria.

[8] Gottschall C, Ferreira E, Canellas L, Bittencourt HR (2008). Perdas reprodutivas e reconcepção em bovinos de corte segundo a idade ao acasalamento. Arq Bras Med Vet Zootec..

[9] Mercadante MEZ, Packer IU, Razook AG, Cyrillo JNSG, Figueiredo LA (2003). Direct and correlated responses to selection for yearling weight on reproductive performance of Nellore cows. J Anim Sci..

[10] Dias LT, El Faro L, Albuquerque LG (2004). Estimativas de herdabilidade para idade ao primeiro parto de novilhas da raça Nellore. Rev Bras Zootec..

[11] Mercadante MEZ, Lôbo RB, Oliveira HN (2000). Estimativas de (co)variâncias entre características de reprodução e de crescimento em fêmeas de um rebanho Nellore. Rev Bras Zootec..

[12] Pereira E, Eler JP, Ferraz JBS (2000). Correlação genética entre perímetro escrotal e algumas características reprodutivas na raça Nellore. Rev Bras Zootec..

[13] Vitezica ZG, Aguilar I, Misztal I, Legarra A (2011). Bias in genomic prediction in populations under selection. Genet Res (Camb).

[14] Maher B (2008). Personal genomes. The case of the missing heritability. Nature.

[15] Peters SO, Kizilkaya K, Garrick DJ, Fernando RL, Reecy JM, Weaber RL (2012). Bayesian genome-wide association analyses of growth and yearling ultrasound measures of carcass traits in Brangus heifers. J Anim Sci..

[16] Varona L, Garcia-Cortes LA, Perez-Enciso M (2001). Bayes factors for detection of quantitative trait loci. Genet Sel Evol..

[17] Habier D, Fernando RL, Kizilkaya K, Garrick DJ (2011). Extension of the Bayesian alphabet for genomic selection. BMC Bioinformatics..

[18] Kass RE, Raftery AE (1995). Bayes factors. J Am Statist Assoc..

[19] Vidal O, Noguera JL, Amills M, Varona L, Gil M, Jiménez N (2005). Identification of carcass and meat quality quantitative trait loci in a Landrace pig population selected for growth and leanness. J Anim Sci..

[20] Espigolan R, Baldi F, Boligon AA, Souza FRP, Gordo DGM, Tonussi RL (2013). Study of whole genome linkage disequilibrium in Nellore cattle. BMC Genomics..

[21] Fortes MRS, Reverter A, Zhang Y, Collis E, Nagaraj SH, Jonsson NN (2010). Association weight matrix for the genetic dissection of puberty in beef cattle. Proc Natl Acad Sci USA.

[22] Fortes MRS, Reverter A, Nagaraj SH, Zhang Y, Jonsson NN, Barris W (2011). A single nucleotide polymorphism-derived regulatory gene network underlying puberty in 2 tropical breeds of beef cattle. J Anim Sci..

[23] Fortes MRS, Li Y, Collis E, Zhang Y, Hawken RJ (2012). The *IGF1* pathway genes and their association with age of puberty in cattle. Anim Genet..

[24] Hawken RJ, Zhang YD, Fortes MR, Collis E, Barris WC, Corbet NJ (2012). Genome-wide association studies of female reproduction in tropically adapted beef cattle. J Anim Sci..

[25] Wathes DC, Clempson AM, Pollot GE (2013). Associations between lipid metabolism and fertility in the dairy cow. Reprod Fertil Dev..

[26] Clempson AM, Pollott GE, Brickell JS, Bourne NE, Munce N, Wathes DC (2011). Polymorphisms in the autosomal genes for mitochondrial function *TFAM* and *UCP2* are associated with performance and longevity in dairy cows. Animal..

[27] Clempson AM, Pollott GE, Brickell JS, Bourne NE, Munce N, Wathes DC (2011). Evidence that leptin genotype is associated with fertility, growth, and milk production in Holstein cows. J Dairy Sci..

[28] Silva JAV, Van Melis MH, Eler JP, Ferraz JBS (2003). Estimação de parâmetros genéticos para probabilidade de prenhez aos 14 meses e altura na garupa em bovinos da raça Nellore. Rev Bras Zootec..

[29] Sahana G, Guldbrandtsen B, Bendixen C, Lund MS (2010). Genome-wide association mapping for female fertility traits in Danish and Swedish Holstein cattle. Anim Genet..

[30] Schulman NF, Sahana G, Iso-Touru T, McKay SD, Schnabel RD, Lund MS (2011). Mapping of fertility traits in Finnish Ayrshire by genome-wide association analysis. Anim Genet..

[31] Pausch H, Flisikowski K, Jung S, Emmerling R, Edel C, Götz KU (2011). Genome-wide association study identifies two major loci affecting calving ease and growth-related traits in cattle. Genetics..

