# Erratum to: Genome-wide association study of reproductive traits in Nellore heifers using Bayesian inference

**DOI:** 10.1186/s12711-015-0150-4

**Published:** 2015-09-17

**Authors:** Raphael B. Costa, Gregório MF Camargo, Iara DPS Diaz, Natalia Irano, Marina M. Dias, Roberto Carvalheiro, Arione A. Boligon, Fernando Baldi, Henrique N. Oliveira, Humberto Tonhati, Lucia G. Albuquerque

**Affiliations:** UNESP, Universidade Estadual Paulista, Faculdade de Ciências Agrárias e Veterinárias, Jaboticabal, 14884-900 São Paulo Brazil

## Erratum to: Genetics Selection Evolution (2015) 47:67 DOI 10.1186/s12711-015-0146-0

After publication of this work in the original article, in response to a comment by a reader of our article who noticed errors on the Manhattan plots, we would like to provide the correct information. On the original paper, the SNP effects were plotted instead of the Bayes factor. Please see Figs. 1 and 2 for the correct information. In addition, we would like to provide the heritability coefficients for age at first calving (0.20) and heifer rebreeding (0.18). We are sorry for any inconvenience.

**Fig. 1:**
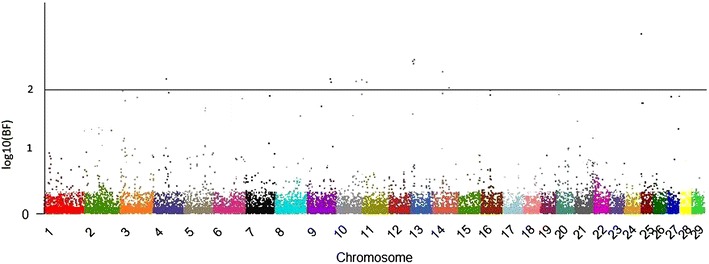
Manhattan plot for age at first calving. The *y*- and *x*-axes indicate the logarithm (base 10) of Bayes factor and chromosome number, respectively

**Fig. 2:**
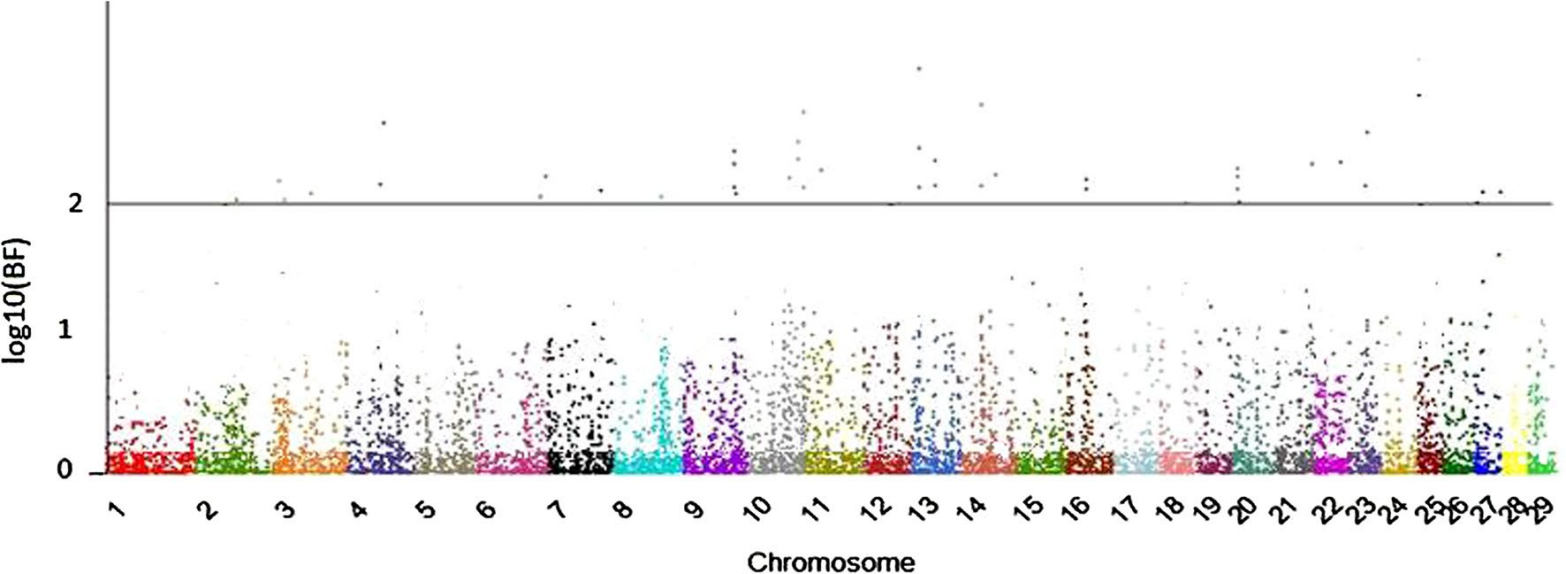
Manhattan plot for heifer rebreeding. The *y*- and *x*-axes indicate the logarithm (base 10) of Bayes factor and chromosome number, respectively

